# The mTORC2 Component Rictor Contributes to Cisplatin Resistance in Human Ovarian Cancer Cells

**DOI:** 10.1371/journal.pone.0075455

**Published:** 2013-09-23

**Authors:** Akechai Im-aram, Lee Farrand, Seung-Min Bae, Gwonhwa Song, Yong Sang Song, Jae Yong Han, Benjamin K. Tsang

**Affiliations:** 1 World Class University Biomodulation Major, Department of Agricultural Biotechnology, College of Agriculture and Life Sciences, Seoul National University, Seoul, Republic of Korea; 2 Departments of Obstetrics & Gynecology and Cellular & Molecular Medicine, and the Interdisciplinary School of Health Sciences, University of Ottawa, Ottawa, Ontario, Canada; 3 Chronic Disease Program, Ottawa Hospital Research Institute, Ottawa, Ontario, Canada; Rush University Medical Center, United States of America

## Abstract

Resistance to cisplatin-based therapy is a major cause of treatment failure in human ovarian cancer. A better understanding of the mechanisms of cisplatin resistance will offer new insights for novel therapeutic strategies for this deadly disease. Akt and p53 are determinants of cisplatin sensitivity. Rictor is a component of mTOR protein kinase complex 2, which is required for Akt phosphorylation (Ser473) and full activation. However, the precise role of rictor and the relationship between rictor and p53 in cisplatin resistance remains poorly understood. Here, using sensitive wild-type p53 (OV2008 and A2780s), resistant wild-type p53 (C13* and OVCAR433), and p53 compromised (A2780cp, OCC1, and SKOV-3) ovarian cancer cells, we have demonstrated that (i) rictor is a determinant of cisplatin resistance in chemosensitive human ovarian cancer cells; (ii) cisplatin down-regulates rictor content by caspase-3 cleavage and proteasomal degradation; (iii) rictor down-regulation sensitizes chemo-resistant ovarian cancer cells to cisplatin-induced apoptosis in a p53-dependent manner; (iv) rictor suppresses cisplatin-induced apoptosis and confers resistance by activating and stabilizing Akt. These findings extend current knowledge on the molecular and cellular basis of cisplatin resistance and provide a rationale basis for rictor as a potential therapeutic target for chemoresistant ovarian cancer.

## Introduction

Epithelial ovarian cancer is the most lethal gynecologic malignancy among women worldwide [1]. Despite advances in our understanding of tumor biology, the overall mortality from ovarian cancer (OVCA) remains high. Currently, chemotherapy in combination with surgical debulking is the preferred treatment option and derivatives of cisplatin (CDDP: cis-diamminedichloroplatinum) are first-line chemotherapeutic therapeutics. CDDP induces cytotoxic cell death through the formation of DNA-platinum adducts, resulting in DNA damage and activation of apoptotic pathways [2].

The effective treatment of OVCA is often hampered by late diagnosis and the emergence of resistance to chemotherapy after successive rounds of treatment. Resistance to chemotherapeutics involves complex mechanisms that can result from dysregulated signaling, enhanced DNA repair, altered cancer cell metabolism [3,4], drug transport and metabolism [5], and the dysregulation of survival factors, including FLIP, Xiap and Akt [6-9]. These molecular and cellular events alter the overall response of the cell to genotoxic agents like CDDP and influence the cell toward pro-survival decisions.

The mammalian target of rapamycin (mTOR) pathway has emerged as a critical regulator of cellular metabolism, growth, proliferation and survival. Its aberration, which is present in up to 50% [10] of OVCA patients, has been shown to confer resistance to CDDP-based treatment and is associated with an adverse prognosis [11-14]. The mTOR pathway involves two signaling complexes: mTORC1 and mTORC2. mTORC1 is sensitive to rapamycin and controls protein synthesis and cellular metabolism, while mTORC2 is essential for cell viability [15]. mTORC2 is also known for its role in the phosphorylation of Akt at Ser473 allowing full activation and proteasomal degradation [16-18]. Akt activation and/or over-expression are a determinant of CDDP sensitivity in human OVCA. Akt activation results in the stabilization of a number of caspase inhibitors [19,20] inhibits mitochondrial p53 accumulation and release of death proteins [21,22], and attenuates p53 phosphorylation and nuclear function [8]. In contrast, Akt inhibition increases p53 phosphorylation (Ser^15^) and CDDP sensitivity [8,23].

The rapamycin-insensitive companion of mTOR (Rictor) is an essential component of the complex mTORC2, and is required for its full function [24]. Over-expression of rictor increases mTORC2 activity and promotes cell growth and motility [25]. Conversely, rictor down-regulation suppresses cell proliferation and tumor formation in certain cancers [26-28]. Rictor also interacts with the integrin-linked kinase (ILK) to promote cancer cell survival through Akt Ser473 phosphorylation, and with PKCζ for cancer cell invasion and metastasis [29,30]. Rictor is required for prostate cancer development induced by PTEN loss [31]. Targeting rictor induces cell cycle arrest at G1 phase and decreases cyclin D1 expression in breast, colon and prostate cancer cells [27,32]. Moreover, down-regulation of mTORC2 facilitates chemotherapeutic drug-induced apoptosis in breast cancer cells [33]. However, the role of rictor in CDDP resistance in OVCA remains unknown.

p53 is a tumor suppressor protein that influences downstream effectors of apoptosis through both transcription-dependent and –independent mechanisms [8,21]. It is normally activated by CDDP via phosphorylation at Ser15 and Ser20, which are essential for its pro-apoptotic properties, and suppression of murine double minute 2 (MDM2) and its ubiquitination and proteasomal degradation [34-36]. We have recently demonstrated that loss of p53 function by inactivation or mutation negatively influences apoptosis and chemosensitivity [7,37]. Cells lacking functional p53 fail to inhibit mTORC1 in response to DNA damage [38]. However, the coordination and communication between p53 status and rictor in the regulation of chemoresistance is poorly understood.

In the present study, we hypothesize that rictor plays an important role in regulating chemosensitivity of OVCA cells and that its down-regulation sensitizes chemoresistant OVCA cells to CDDP treatment by facilitating Akt-dependent proteasomal degradation, in a manner dependent upon p53 status. The outcomes of this study raise the possibility that rictor may be a therapeutic target for OVCA, although the effectiveness of such an application is likely to be dependent on p53 status.

## Materials and Methods

### Reagents

RPMI 1640 and DMEM/F12 culture media, fetal bovine serum (FBS), non-essential amino acids, penicillin, streptomycin, and amphotericin B were from Life Technologies (Carlsbad, CA, USA). CDDP, dimethyl sulfoxide (DMSO), Hoechst 33248, aprotinin, sodium orthovanadate (Na _3_VO_4_), phenylmethylsulfonyl fluoride (PMSF) were from Sigma-Aldrich (St. Louis, MO, USA). Rabbit polyclonal antibodies: anti-phospho-Ser^473^-Akt, anti-phospho-Ser^450^-Akt, anti-Akt, anti-phospho-Ser^15^-p53, anti-PARP and Rabbit monoclonal anti-rictor antibody were purchased from Cell Signaling Technology (Beverly, CA, USA). Mouse monoclonal anti-p53 and anti-GAPDH antibodies were from Santa Cruz Biotechnology (Santa Cruz, CA, USA) and Abcam (Cambridge, MA, USA), respectively. Goat anti-mouse and anti-rabbit secondary antibodies were from Bio-Rad Laboratories (Hercules, CA, USA). Rictor and p53 siRNA were purchased from Cell Signaling Technology (Beverly, CA, USA). Control siRNA was from Santa Cruz Biotechnology (Santa Cruz, CA, USA). Lipofectamine 2000, RNase A, N,N,N',N'-tetramethyl-ethane-1,2-diamine (TEMED), TRIzol and ROX dye were from Invitrogen (Carlsbad, CA, USA). RNeasy Mini Kit was from Qiagen (Valencia, CA, USA). Adenovirus constructs containing the wt-p53 and eGFP transcripts were from Vector Biolabs (Philadelphia, PA, USA). Epoxomycin and lactacystin were from EMD Chemical (Gibbstown, NJ, USA). Z-DEVD-FMK and Z-VAD-FMK were from Tocris Bioscience (Ellisville, MO, USA). Rictor and GAPDH RNA primer were from Bioneer (Daejeon, Korea) and human recombinant active caspase 3 was from BioVision (Mountain View, CA, USA). PIPES, DL-Dithiothreitol (DDT), Ethylenediamine-tetraacetic Acid (EDTA) and 3[(3-cholamido-propyl) dimethyallonio]-1-propanesulfonate hydrate (Chaps) were purchased from Sigma-Aldrich (Saint Louis, MO, USA).

### Cell lines and Culture

CDDP sensitive (OV2008 and A2780s) and resistant (C13*, OVCAR-433, A2780cp, OCC-1, and SKOV3) human OVCA cell lines were generously provided by Drs. Rakesh Goel and Barbara Vanderhyden (Ottawa Hospital Cancer Center, Ottawa, ON, Canada). The cells were maintained in RPMI 1640 and DMEM/F-12 as previously reported [20,21,36]. The OV2008 cell line and its resistant counterpart C13* were originated from ovarian endometrioid adenocarcinoma with squamous differentiation. OCC-1, A2780s and A2780cp cells originated from undifferentiated ovarian carcinoma tumors. SKOV3 cells were of a clear cell carcinoma origin [39] and OVCAR433 cells were of serous cystadenocarcinomas of the ovary [23]. Following the indicated treatments, the cells were harvested for analysis.

### Protein extraction and Western blotting analysis

The procedures for protein extraction and Western blotting analysis were performed as previously described [40]. Membranes were incubated at 4°C overnight with anti-rictor (1:100), phospho-Ser^473^-Akt (1:1000), phospho-Ser^450^-Akt (1:1000), p53 (1:5000), phospho-Ser^15^-p53 (1:1000) and PARP (1:2000) antibodies and 1 h at room temperature for anti-GAPDH (1:10,000) and HRP-conjugated rabbit or mouse secondary antibodies (1:5000-1:10,000). GAPDH was chosen as loading control since its cellular content in the OVCA cells examined in the current studies is not affected by CDDP treatment. Band densities were analyzed for quantification using a ChemiDOC^TM^ XRS+ (Bio-Rad Laboratories, Hercules, CA, USA)

### Assessment of Apoptosis

Apoptosis was assessed based on cellular morphology using the nuclear stain Hoechst 33258. The procedure was performed as previously described [20,36]. A minimum of 400 cells per treatment group were counted and the counter was blinded to avoid experimental bias.

### siRNA transfection

OVCA cells were transfected with rictor siRNA (0-100 nM; 48 h), p53 siRNA (100 nM; 48 h), or control siRNA (0-100 nM; 48 h) and treated thereafter with CDDP (0-10 µM; 24 h), as previously described [20], and harvested for further analysis.

### Adenoviral infection

A2780cp and SKOV3 cells were infected with adenoviral wt-p53 (MOI = 10; 24 h; GFP as control) as previously described [9,20].

### In Vitro caspase-3 activity

The in vitro caspase-3 activity assay was performed with whole cell lysates of OV2008 as previously described [23].

### Statistical analysis

Results are expressed as the mean ± SEM of at least three independent experiments. Statistical analysis was performed by paired t-test, and one-way or two-way ANOVA as appropriate, using SigmaPlot® 12 (Systat Software Suite, IL, USA). Differences between multiple experimental groups were determined by the Bonferroni post-hoc test. Statistical significance was inferred at P < 0.05.

## Results

### CDDP down-regulates Rictor content and induces apoptosis in chemosensitive but not resistant OVCA cells

**Figure 1:**
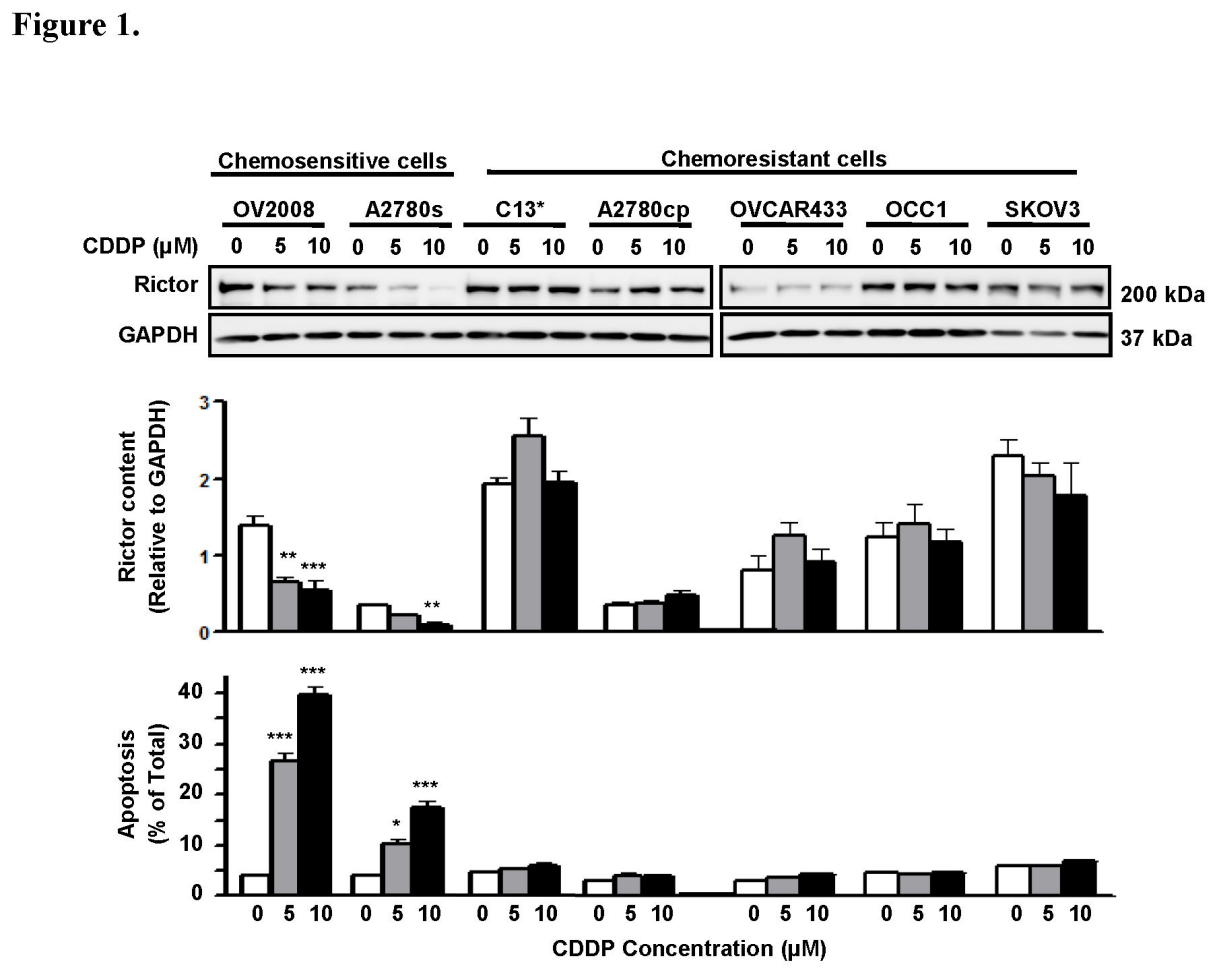
CDDP down-regulates rictor content and induces apoptosis in chemosensitive but not resistant OVCA cells *in vitro*. Rictor protein expression is down-regulated in chemosensitive (OV2008 and A2780s) but not chemoresistant OVCA (C13* and A2780cp*) during CDDP treatment (0-10 µM CDDP; 24 h). Decreased rictor content in OV2008 and A2780s following CDDP treatment was associated with increased apoptosis. Rictor protein content in chemoresistant OVCA (OVCAR433, OCC1 and SKOV3) was not affected by CDDP. Rictor content was normalized against GAPDH (loading control). A representative Western blot from three independent experiments is shown. Results are presented as mean ± SEM of three independent experiments. *p<0.05, **p<0.01, ***p<0.001, #p<0.05 (vs CTL in sensitive cells). Hoechst 33258 staining was used for assessment of apoptosis as mentioned in experimental procedures.

To determine whether rictor content is altered during CDDP treatment in OVCA, chemosensitive OVCA (OV2008 and A2780s) and chemoresistant (C13*, A2780cp*, OVCAR433, OCC1 and SKOV-3) OVCA cell lines were treated with CDDP (0-10 µM; 24 h) and rictor content was assessed by immunoblotting [Figure 1]. CDDP significantly down-regulated intact rictor content (200 kDa) and induced apoptosis in chemosensitive (OV2008, ***P<0.001; and A2780s, **P<0.01), but not in chemoresistant OVCA irrespective of CDDP concentration (C13*, A2780cp, OVCAR433, OCC1 and SKOV-3; p>0.05). These results demonstrate that rictor is not subjected to down-regulation by CDDP in chemoresistant OVCA cells, a phenomenon that may contribute to the resistant phenotype.

### CDDP induces rictor processing and chemosensitivity in a caspase-3- and proteasome-dependent manner

**Figure 2:**
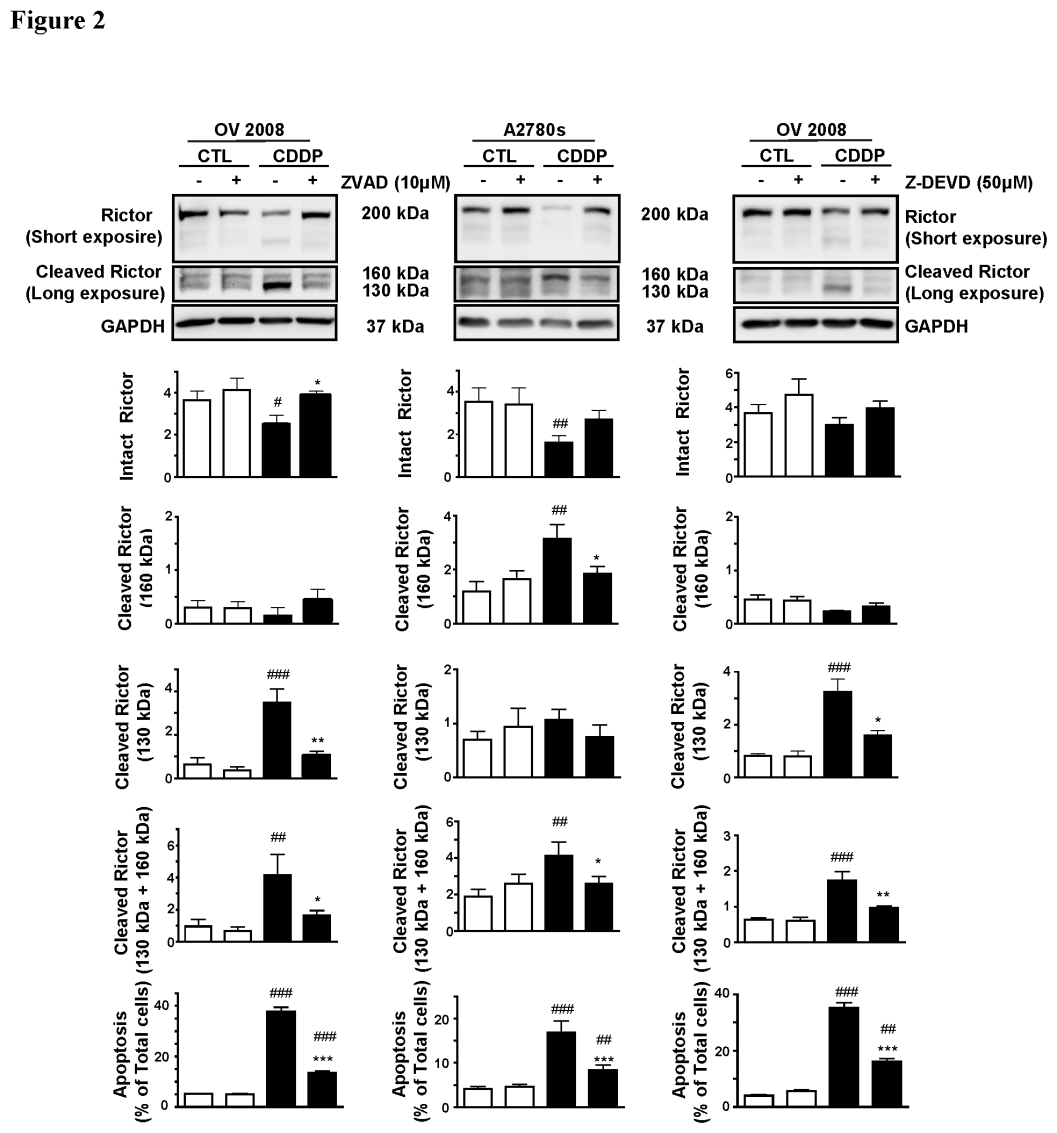
CDDP-induced rictor downregulation in CDDP-sensitive cells involves caspase-3-mediated cleavage. OV2008 and A2780s were pretreated with Z-VAD FMK (10 µM) and Z-DEVD FMK (50 µM) for 30 minutes before and during CDDP challenge (0-10 µM; 24 h) and rictor content and apoptosis were assessed. OV2008 cells treated with CDDP alone exhibited an intact rictor (200 kDa) and two cleaved products (160 kDa and 130 kDa). CDDP decreased intact rictor content and 160 kDa protein but markedly increased levels of the 130 kDa band. Treatment of A2780s with CDDP resulted in down-regulation of intact rictor but increased the level of the 160 kDa protein and had no effect on the 130 kDa protein. Pre-treatment of the cells with the pan-caspase inhibitor (Z-VAD) or the specific caspase-3 inhibitor (Z-DEVD) significantly attenuated the CDDP-induced changes in intact and cleaved rictor contents in both sensitive cells, and CDDP-induced apoptosis was significantly but not completely attenuated by the presence of the inhibitors in both chemosensitive cell lines. Rictor content was normalized against GAPDH (loading control). Results are presented as mean ± SEM (n=3 and n=5 in OV2008 and in A2780s, respectively). *p<0.05, **p<0.01, ***p<0.001 (vs respective controls). Hoechst 33258 staining was used for assessment of apoptosis as mentioned in experimental procedures.

To determine if CDDP-induced rictor down-regulation could be due to post-translational processing, we first investigated the possibility of caspase-dependent cleavage of rictor in OV2008 and A2780s when treated with the pan-caspase inhibitor Z-VAD FMK (10 µM) before (30 min) and during CDDP challenge (0-10 µM; 24 h). OV2008 cells treated with CDDP alone exhibited an intact rictor (200 kDa) and two immunoreactive cleaved products which migrated at 160 kDa and 130 kDa [Figure 2]. CDDP decreased intact rictor content and the 160 kDa protein but markedly increased the levels of 130 kDa band. Although treatment of A2780s with CDDP also resulted in down-regulation of intact rictor (200 kDa), the level of the 160 kDa protein was markedly elevated while that of the 130 kDa was not significant affected. Pre-treatment of the cells with the caspase inhibitor significantly attenuated the CDDP-induced changes in intact and cleaved rictor contents in both sensitive cells (P<0.05 and P<0.01 in OV2008 and A2780s, respectively, Figure 2), suggesting that CDDP down-regulates rictor in part by increased caspase activity and that cleavage at different caspase consensus sites may be involved. In addition, pretreatment of the OVCA cells with the specific caspase-3 inhibitor Z-DEVD-FMK produced similar results, indicating that caspase-3 is involved in CDDP-induced rictor processing. Interestingly, while CDDP induced apoptosis in both chemosensitive cell lines, pretreatment of the cells with either the pan-caspase or specific caspase-3 inhibitor attenuated this response, suggesting that CDDP induces rictor processing by caspase-3 and dysregulation of this process confers chemoresistance in OVCA cells.

**Figure 3:**
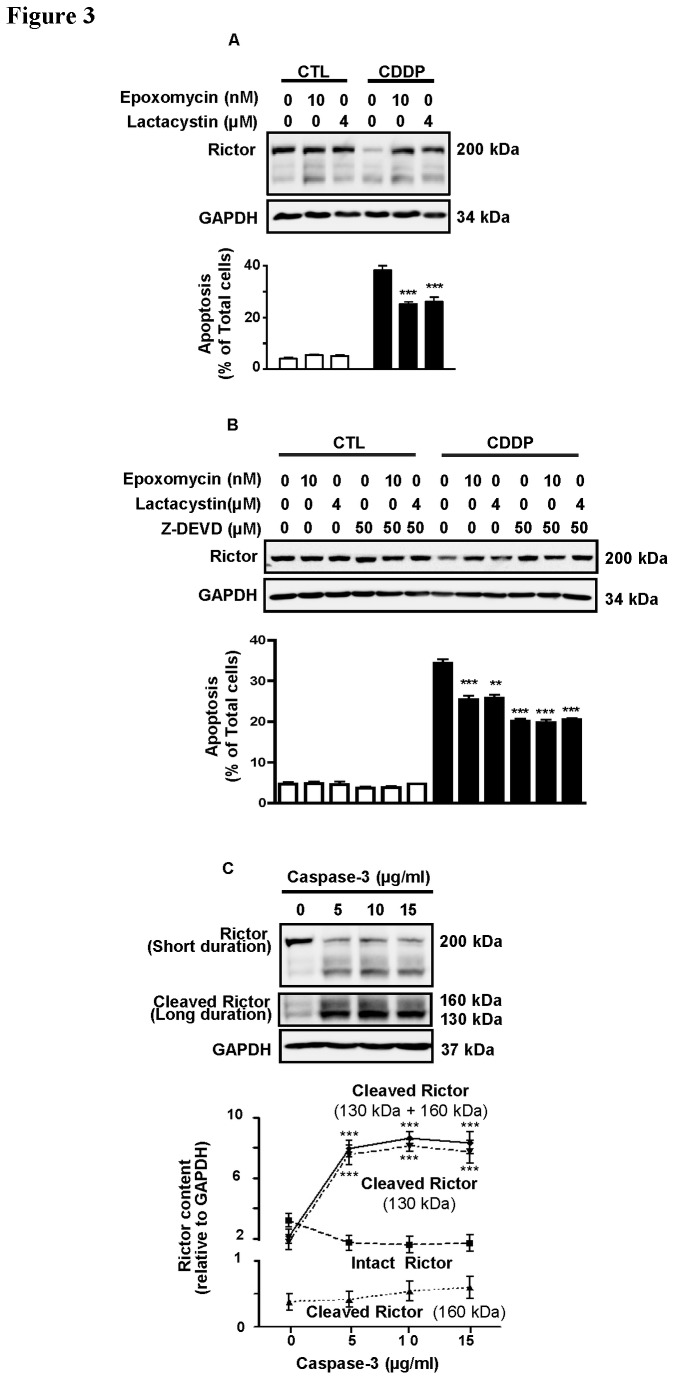
Proteasome-mediated degradation and caspase-3 activity are responsible for CDDP-induced rictor down-regulation in chemosensitive OVCA. **A**. OV2008 cells were pretreated (30 min) with the proteasomal inhibitors [epoxomycin (10 nM) and lacytasystin (4 µM)] and subjected to CDDP challenge (0-10 µM; 24 h). Rictor content and apoptosis were analyzed by Western blotting and nuclear morphology assessment. Both epoxomicin and lactacystin effectively blocked CDDP-induced rictor degradation (P<0.001) but only partially attenuated apoptosis. **B**. OV2008 cells were cultured in the same conditions as in A, but pretreated with proteasome inhibitor and/or Z-DEVD (50 µM). No synergistic effect between the two inhibitors was observed. **C**. Incubation of OV2008 whole cell lysate (30 min, 30°C) with recombinant active caspase-3 (5-20 µg/ml) resulted in Rictor cleavage as evidenced by decreased intact rictor content (200 kDa) and increased in the cleaved forms (130 kDa and 160 kDa). Results are presented as mean ± SEM of three independent experiments. *p<0.05, **p<0.01, ***p<0.001 (vs respective controls). Hoechst 33258 staining was used for assessment of apoptosis as mentioned in experimental procedures.

To determine whether proteasomal degradation plays a role in CDDP-induced rictor down-regulation, OV2008 cells were cultured [30 min pre-treatment; 24 h during treatment with CDDP (0-10 µM)] with the proteasome inhibitors epoxomycin (10 nM) and lactacystin (4 µM). Whereas CDDP alone significantly down-regulated intact rictor and induced apoptosis, as expected, the presence of the inhibitors completely blocked CDDP-induced rictor down-regulation and significantly, but not completely attenuated apoptosis, as evidenced by PARP cleavage and nuclear morphology (P<0.001; Figure 3: A).

We then investigated further if rictor processing by caspase-3 activity and proteasomal degradation occurs in separate pathways. OV2008 cells were cultured [30 min pre-treatment; 24 h during treatment with CDDP (0-10 µM)] with proteasome inhibitors and/or specific caspase-3 inhibitor. The same results were observed when either inhibitor was present. However, no additional effect was observed when both inhibitors were used together [Figure 3: B].

In addition, to provide further evidence that rictor processing induced by CDDP treatment is caspase-3 dependent, whole cell lysates from OV2008 cells were used to perform an *in vitro* caspase-3 activity assay. The *in vitro* data was in agreement to that obtained from the cell line experiment [Figure 3: C]. Taken together, these results indicate that CDDP down-regulates intact rictor at the protein level via caspase-3 cleavage and proteasomal degradation.

### Rictor knockdown sensitizes chemo-resistant OVCA to CDDP-induced apoptosis

**Figure 4:**
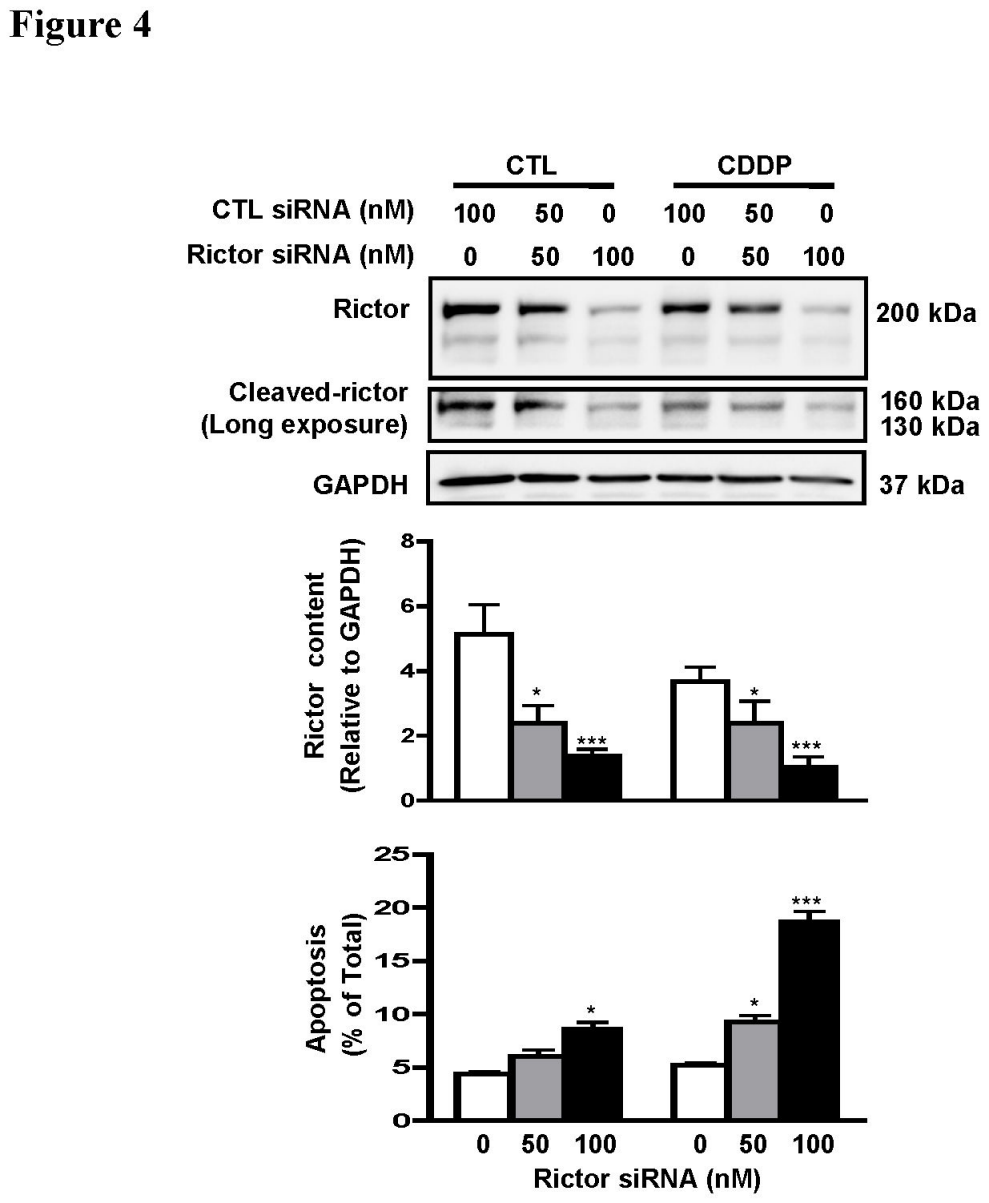
Rictor knockdown sensitizes chemoresistant OVCA cells to CDDP-induced apoptosis. C13* cells were transfected with rictor siRNA (0-100 nM; 48 h) and cultured with or without CDDP (10 µM; 24 h). Rictor knockdown significantly enhanced CDDP-induced apoptosis in C13* cells in a concentration-dependent manner. Results are expressed as mean ± SEM of three independent experiments. *p<0.05, ***p<0.001 (vs respective CTL siRNA). Hoechst 33258 staining was used for assessment of apoptosis as mentioned in experimental procedures.

To establish the role of rictor in OVCA chemoresistance, rictor expression in a wt-p53 chemoresistant OVCA cell line (C13*) was silenced by siRNA (0, 50 and 100 nM; 48 h) prior to treatment with CDDP (10 µM; 24 h), and apoptosis was assessed. Intact rictor and its cleaved forms were significantly down-regulated by siRNA in a concentration-dependent manner. A significant decrease in intact rictor and cleaved rictor were observed at 50 nM (p<0.05) and to an approximate 30% reduction at 100 nM, irrespective of the presence of CDDP. These findings not only confirmed that the 160kDa and 130 kDa immunoreactive bands were indeed cleaved products of rictor, but also demonstrated that rictor knockdown induced apoptosis (p<0.05) as well as enhanced CDDP-induced apoptosis in a concentration-dependent manner (p<0.001) [Figure 4]. These results suggest that rictor is an important determinant of CDDP resistance in OVCA.

### Apoptotic response to CDDP following rictor down-regulation is dependent on p53 status

**Figure 5:**
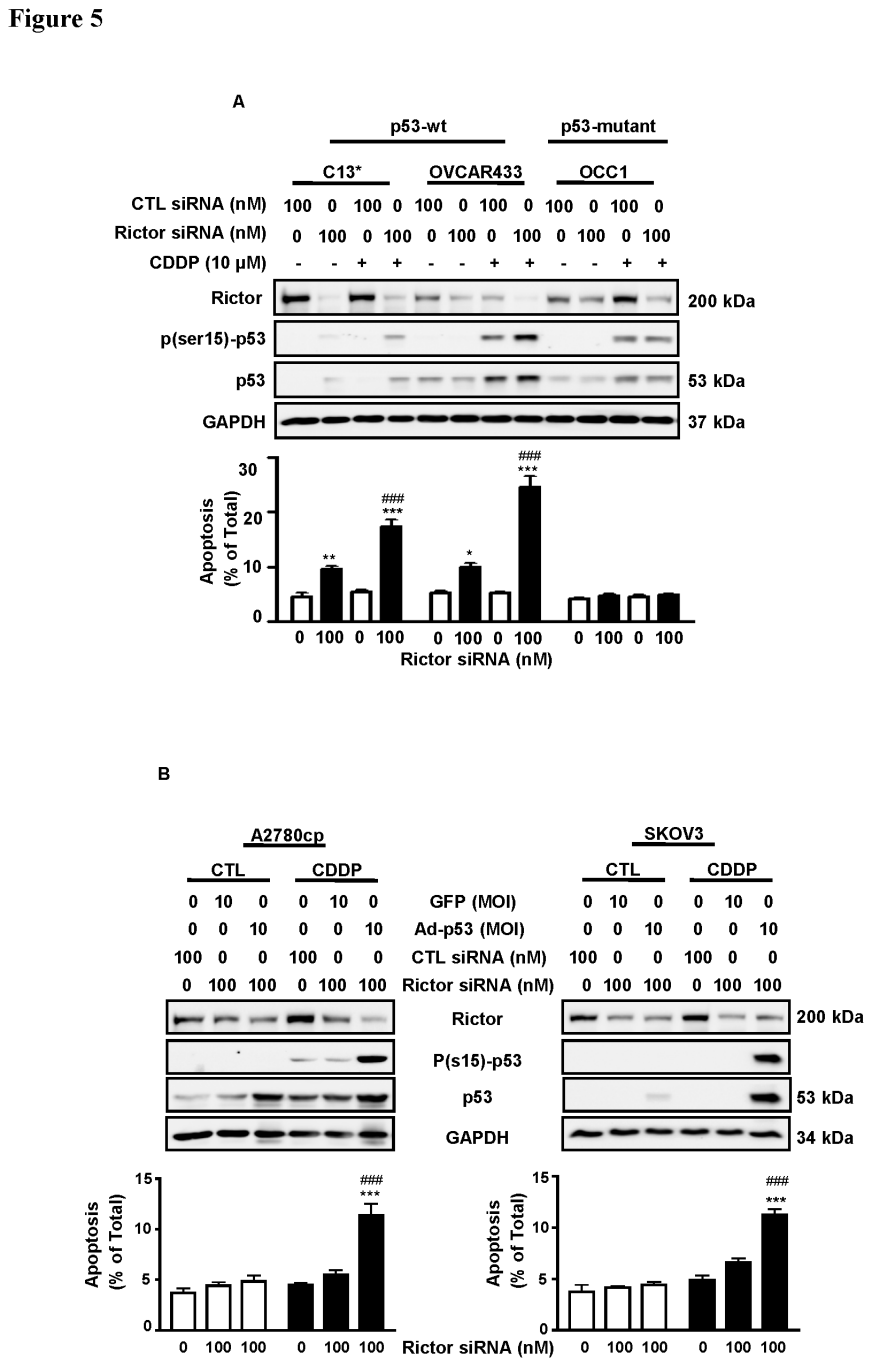
Apoptotic response of chemoresistant OVCA cells to CDDP following rictor down-regulation is dependent on p53 status. Chemoresistant OVCA cell lines with varying p53 status (wt-p53, C13* and OVCAR433; p53-mutant, OCC1 and A2780cp; p53-null, SKOV3) were transfected with rictor siRNA (100 nM siRNA, 48 h) with or without adenoviral wt-p53 infection (0-10 MOI; 24 h; p53-deficient cells) and CDDP treatment (0-10 µM CDDP; 24 h). Rictor, p-p53 ser15, p53, PARP and GAPDH content, as well as apoptosis were assessed. Rictor knock-down significantly sensitized chemoresistant wt-p53 cells (**A**, C13* and OVCAR433; P<0.001) but not p53-difficient cells (**A**, OCC1; **B**, A2780cp and SKOV3) to CDDP (10 µM)-induced apoptosis. Reconstitution in A2780cp and SKOV-3 of wt-p53 significantly enhanced CDDP-induced apoptosis (P<0.001). Results are presented as mean ± SEM of three independent experiments. *p<0.05, **p<0.01, ***p<0.001 (vs respective CTL siRNA), # # #p<0.05 (vs controls Adenoviral infection). Hoechst 33258 staining was used for assessment of apoptosis as mentioned in experimental procedures.

**Figure 6:**
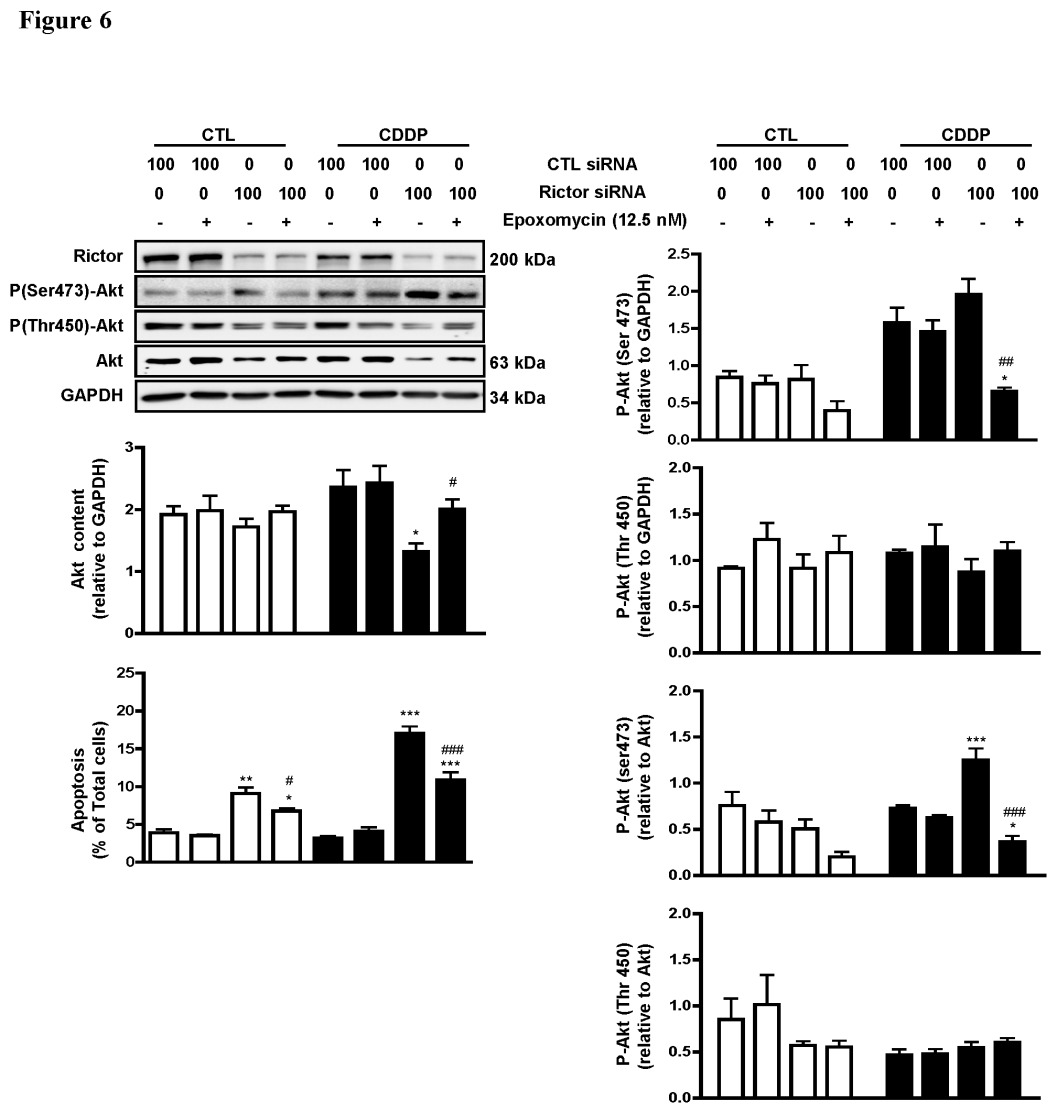
Rictor down-regulation sensitizes chemoresistant OVCA cells to CDDP-induced apoptosis by facilitating Akt proteasomal degradation. Chemoresistant OVCA cells (C13*) were transfected with rictor siRNA (100 nM siRNA, 48 h) and pretreated with epoxomycin (0-12.5 nM) 30 minutes prior to CDDP treatment (0-10 µM CDDP; 24 h). Rictor, Akt, phospho- Akt (Thr450 and Ser473), and GAPDH content, as well as apoptosis were assessed. Rictor knock-down significantly enhanced CDDP-induced apoptosis in chemoresistant OVCA cells (P<0.01 and P<0.001, respectively) and this effect was rescued by epoxomycin (P<0.05 and p<0.001, respectively) Total-Akt was significantly decreased during rictor silencing with CDDP treatment (P<0.05) and a massive increase in the ratio between phospho-Akt (Ser473) and total-Akt was also observed, which significantly decreased when epoxomycin was added (P<0.001 and P<0.05, respectively) Results are presented as mean ± SEM of three independent experiments. *p<0.05, **p<0.01, ***p<0.001 (vs respective CTL siRNA), # #p<0.01, # # #p<0.05 (vs without epoxomycin). Hoechst 33258 staining was used for assessment of apoptosis as mentioned in experimental procedures.

The tumor suppressor p53 is an important mediator of CDDP-induced apoptosis [9,22]. Since approximately 50% of OVCA patients carry *TP53* gene mutation(s) [41], it is of interest to determine if CDDP-induced apoptosis in chemoresistant cells following rictor knockdown is dependent on the presence of a functional p53. To investigate this possibility, rictor expression in chemoresistant OVCA cell lines with varying p53 status (wt-p53, C13* and OVCAR433; p53-mutant, OCC1 and A2780cp; p53-null, SKOV3) were silenced with rictor siRNA (100 nM siRNA, 48 h) prior to treatment with CDDP (0-10 µM, 24 h). Rictor knock-down significantly sensitized chemoresistant wt-p53 cells (C13* and OVCAR433, Figure 5: A; P<0.001) but not p53-deficient cells (OCC1, Figure 6: A; A2780cp and SKOV3, Figure 5: B) to CDDP-induced apoptosis, suggesting that a functional p53 might be needed for CDDP-induced apoptosis in the chemoresistant OVCA cells with rictor knockdown. To further investigate this hypothesis, CDDP-resistant OVCA cell lines harboring a p53 mutation (A2780cp) and a p53-null line (SKOV-3) were treated with rictor siRNA (0-100 nM; 48 h), followed by reconstitution of wt-p53 via adenoviral infection (0-10 MOI; 24 h) and CDDP treatment (0-10 µM CDDP; 24 h). As shown in Figure 5: B, while rictor knockdown alone did not significantly increase CDDP sensitivity in the absence of wt-p53, wt-p53 reconstitution significantly enhanced the effects of rictor-knockdown on CDDP-induced phospho-p53 Ser15 content and apoptosis in both p53-deficient cell lines (P<0.001).

### Rictor down-regulation sensitizes chemoresistant cells to CDDP-induced apoptosis by facilitating Akt proteasomal degradation

Rictor is known to be required for mTORC2 to phosphorylate Akt at Ser473. To examine if and how Akt is relevant to this phenomenon, chemoresistant OVCA cells (C13*) rictor expression was silenced by siRNA (100 nM; 48 h) prior to pre-treatment with epoxomycin (12.5 nM; 30 min) and treatment with CDDP (10 µM; 24 h), and apoptosis was assessed. Rictor knockdown alone increased phospho-Akt (Ser473) content but decreased the levels of phospho-Akt Ser450 [Figure 6], a phosphorylation site known to be regulated in Akt stability [18]. The increase in Akt phosphorylation at Ser473 following rictor silencing without the proteasomal inhibitor was accompanied by Akt down-regulation (P<0.05 in treatment group) and was rescued by proteasomal inhibitor (p<0.05). Apoptosis induction irrespective of the presence of CDDP, two responses attenuated by the presence of the proteasome inhibitor epoxomycin (Figure 6; P<0.05 and P<0.001 in control and CDDP treatment groups respectively). Moreover, the ratio between phospho-Akt at Ser473 and total Akt was also markedly increased in rictor knockdown with CDDP-treatment and significantly decreased when epoxomicin was added (P<0.001 and p<0.05; with and without epoxomycin, respectively). These results suggest that rictor plays a role in Akt stabilization and CDDP resistance in human OVCA and its knockdown promotes Akt proteasomal degradation and enhances CDDP sensitivity.

## Discussion

Chemoresistance is a major therapeutic hurdle in ovarian cancer and its cellular mechanism is complex and poorly understood. Although the mTOR signaling pathway is known to promote cell proliferation and survival, whether rictor is involved in the control of chemosensitivity is unknown. In the present study, we have investigated the role of rictor in CDDP resistance using CDDP-sensitive and resistant OVCA cell lines with different p53 status. We have demonstrated for the first time that rictor is a determinant of CDDP resistance in OVCA. CDDP down-regulates rictor content in chemosensitive cells by caspase-3 and proteasome-dependent degradation, but no similar effect was observed in resistant cells. In addition, we have shown that rictor suppresses CDDP-induced apoptosis and confers resistance by activating and stabilizing Akt. Silencing rictor sensitizes chemoresistant OVCA cells to CDDP-induced apoptosis in cells harboring wild type-p53, but not in p53-compromised cells unless reconstituted with a functional p53. Taken together, these data support the hypothesis that rictor down-regulation sensitizes chemoresistant OVCA cells to CDDP treatment by facilitating Akt-dependent proteasomal degradation, in a manner dependent upon p53 status.

Our present investigation shows that rictor is down-regulated at protein but not mRNA level (data not shown) and this process is associated with CDDP-induced apoptosis in sensitive OVCA cells and that rictor plays a role in cell fate determination. This observation is further supported with results using various chemoresistant OVCA cell lines with different p53 status, in that the failure of CDDP to down-regulate rictor content allows the cells to survive CDDP treatment. The role of rictor in cell survival has previously been demonstrated, and one study has addressed the notion that rictor and proteins in the mTOR pathway are down-regulated via the proteasome-ubiquitin pathway in lung cancer cells after chemotherapeutic challenge, the possible involvement of rictor processing in the control of chemosensitivity was not addressed [26]. Our results showing that CDDP induces rictor down-regulation via the proteasome are in agreement with this finding. Moreover, our present studies have extended the above findings by showing that caspase-3 is required for CDDP –induced rictor processing in chemosensitive human OVCA cells. Taken together, these results suggest that rictor confers CDDP resistance in human OVCA and CDDP-induced rictor down-regulation is dependent upon both caspase-3 activity and proteasomal degradation in chemosensitive, but not in chemoresistant OVCA cell lines.

Tumor suppressor *TP53* is also one of the most frequently mutated genes in human OVCA, and could be as high as 50% in OVCA patients [41]. Loss or mutation of p53 can have an enormous impact on tumor aggressiveness, prognosis and successful implemention of treatment [42]. Since the mechanism of CDDP-resistance is multifactorial and p53 status is one of the major concerns in human OVCA, we have investigated the relationship between rictor and the tumor suppressor p53, in the aspect of chemoresistance. Our investigation, therefore, further addresses the role of rictor in CDDP resistance and shows that rictor knockdown not only promotes apoptosis, but also enhances CDDP-induced apoptosis in human OVCA cells. This result correlates with a recent study showing that targeting of rictor prevents cell migration and promotes apoptosis in breast cancer [23]. Results from our current study also show that sensitizing chemoresistant OVCA cells by silencing rictor appears to depend upon p53 status. This notion is further supported by the observation that a combination of rictor knockdown and reconstitution of wt-p53 enhances apoptosis in p53-compromised cells when induced by CDDP treatment, an outcome that does not eventuate without wt-p53. The latter response is concomitant with an increase in p53 activation through phosphorylation at the ser15 residue, which changes p53 structure and inhibits the p53-MDM2 interaction, thereby stabilizing p53 [35]. This could be the reason for p53 stabilization observed in A2780cp and SKOV-3 in the presence of phospho-p53 (ser15). Taken together, these observations suggest that rictor knockdown induces apoptosis and enhances CDDP-induced apoptosis in a p53-dependent manner.

The Akt signaling pathway is a determinant of chemoresistance and its activation promotes a chemoresistant phenotype, frequently detected in as high as 87% of cases in human OVCA [10]. The interplay between Akt and mTOR complexes is essential for their cellular function and rictor is known to be required for phosphorylation of Akt at both the hydrophobic motif (Ser473) and the turn motif (Thr450), allowing full activation and triggering Akt to proteasomal degradation, respectively [17,18,43]. In the present study, we have illustrated that rictor contributes to CDDP resistance in human OVCA, in part by activating and stabilizing Akt. Silencing rictor sensitizes human OVCA cells to CDDP-induced apoptosis through facilitating Akt proteasomal degradation. Although our findings are in contrast to previous studies showing that rictor knockdown attenuates Akt phosphorylation at Ser473 and function [27,31,33], mTORC2 disruption has been reported to destabilize Akt due to its ability to phosphorylate Akt at Ser 473 but not at Thr450, thus leading to preferential degradation and inactivation [18,44]. These notions are also supported by the further observation that Akt and phospho-Akt (Thr450) content were decreased, consistent with an increase in phospho-Akt (Ser473), when rictor expression was silenced. This phenomenon could be rescued by a proteasomal inhibitor. Although significantly increased levels of phospho-Akt (Ser473) were not observed after rictor silencing with CDDP treatment, that total-Akt content was decreased significantly could be a reason. This observation was further buttressed by the massive increase in the ratio between phospho-Akt (Ser473) and total-Akt when treated with CDDP with rictor knockdown. Conversely, phospho-Akt (Thr450) was much higher in content in the cells without rictor knockdown, resulting in Akt stabilization and promoting CDDP resistance. Another observation is that phospho-Akt (Thr450) content was intimately related to total-Akt content. This could indicate that phosphorylation of Akt at Ser473 is a predominant mechanism steering Akt stability and degradation in human OVCA. Rictor induced and enhanced CDDP-induced apoptosis which was also attenuated by Akt stabilization following epoxomycin treatment. Taken together, our findings suggest that rictor is indispensable for mTORC2 to stabilize and activate Akt and expands the current knowledge that rictor knockdown not only attenuates Akt function, but also facilitates proteasomal degradation.

**Figure 7:**
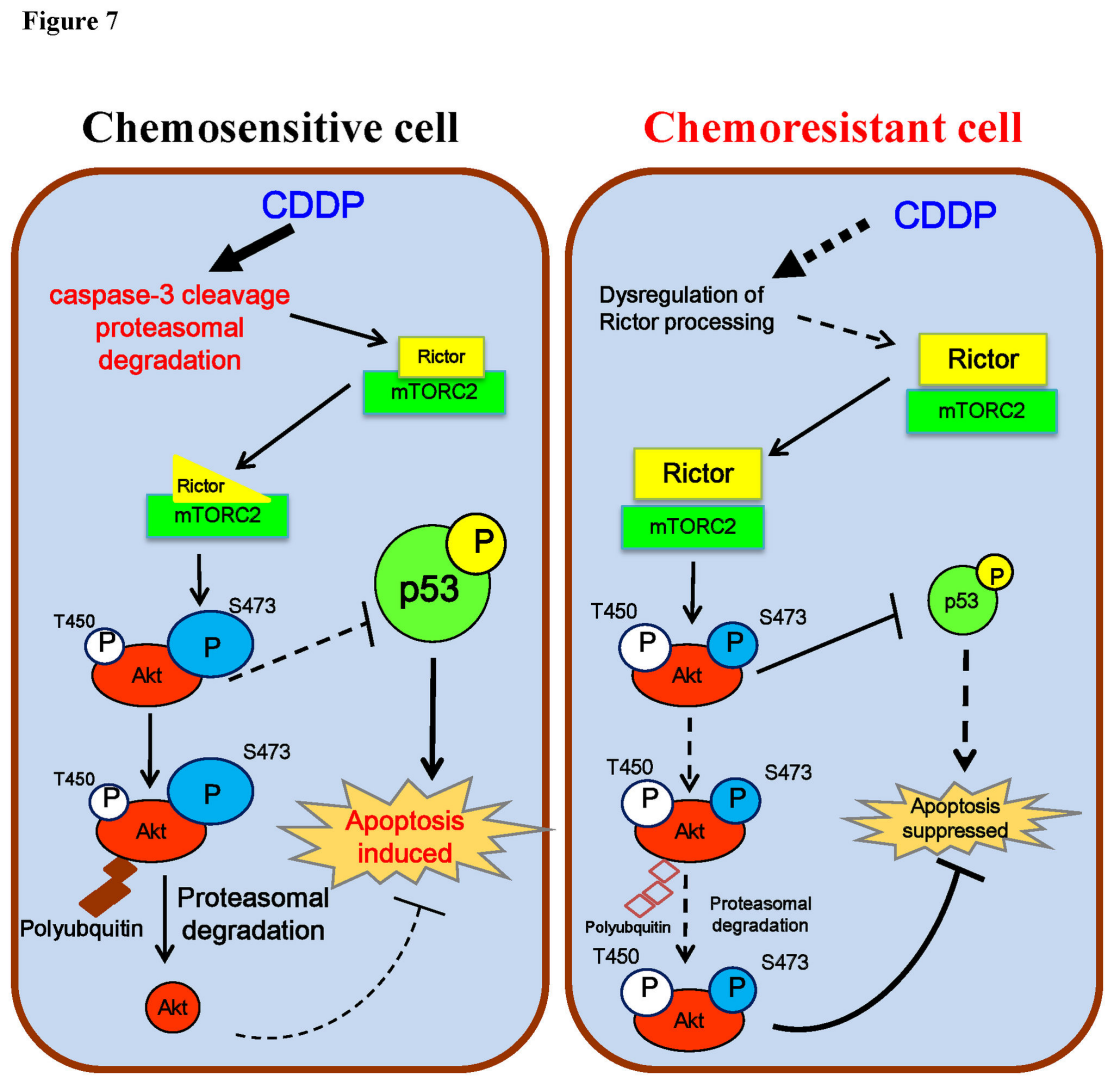
A hypothetical model illustrating the role of rictor in regulation of CDDP sensitivity in OVCA cells. In chemosensitive cells, CDDP activates caspase-3 and induces proteasomal degradation for rictor processing, and consequently destabilizes mTORC2, The unstable mTORC2 complex then facilitates Akt phosphorylation at Ser473, an event known to promote Akt proteasomal degradation and the induction of apoptosis. However, in chemoresistant cells, high level of stabilized rictor promotes Akt activation and stabilization, thereby contributing to CDDP resistance.

In summary, we have demonstrated for the first time that CDDP-induced rictor down-regulation involves caspase-3 cleavage and proteasomal degradation, and that rictor plays a role in chemoresistance by promoting Akt activation and stabilization in OVCA. This occurs in a p53-dependent manner. To facilitate our future investigation on the precise role of rictor in CDDP resistance in OVCA cells, we have proposed a hypothetical model [Figure 7]. In chemosensitive cells, CDDP activates caspase-3 and induces proteasomal degradation for rictor processing, and consequently destabilizes mTORC2, the unstable mTORC2 complex then facilitates Akt phosphorylation at Ser473, an event known to promote Akt proteasomal degradation and the induction of apoptosis. However, in chemoresistant cells, high level of stabilized rictor promotes Akt activation and stabilization, thereby contributing to CDDP resistance. However, precisely how mTORC2 regulates Akt phosphorylation and its site-specificity in the control of chemosensitivity in ovarian cancer cells and whether DNA-dependent protein kinase is also involved, remains to be determined. The mechanism(s) by which caspase-mediated processing and proteasomal degradation of rictor is regulated and the interdependence of these processes also require further investigation. Although Akt has been shown to be ubiquitinated and degraded by the proteasome upon phosphorylation at Ser 473 in both normal and malignant cells [18], the molecular mechanism involved in Akt processing in OVCA needs to be further demonstrated. Moreover, changes in the half-life of Akt and its downstream events in response to CDDP treatment and proteasomal inhibition needs to be assessed so as to provide greater insights into the consequences of Akt degradation and its influence on chemosensitivity in OVCA cells. Establishing the role of rictor in chemoresistance may justify the targeting of rictor as a novel therapeutic strategy in overcoming CDDP resistance in human OVCA, particularly for those with wt-p53 status. Targeting rictor may potentiate effective therapeutic outcomes, since this is likely to not perturb the negative feedback pathway between Akt and mTORC1. However, our findings need to be verified *in vivo* to support the validity of this hypothesis.
